# Fibroelastoma, uma Doença *Incidentaloma*? – Imagem de Casos de Fibroelastomas como Achados Incidentais em Quatro Pacientes, Quatro Valvas Diferentes

**DOI:** 10.36660/abc.20230222

**Published:** 2024-02-16

**Authors:** Joana Lima Lopes, Antonio Freitas, João Bicho Augusto

**Affiliations:** 1 Hospital Prof. Doutor Fernando Fonseca Amadora Portugal Hospital Prof. Doutor Fernando Fonseca, Amadora – Portugal

**Keywords:** Fibroelastoma, Achados Incidentais, Neoplasias Cardíacas/cirurgia, Valvas Cardíacas, Ecocardiografia Transesofagiana/métodos, Embolização

## Abstract

Os fibroelastomas são o segundo tumor cardíaco benigno mais comum. São estruturas pequenas, avasculares, com uma dimensão média de 9mm, podendo atingir até 70mm, habitualmente aderentes à superfície das válvulas cardíacas (válvulas aórtica e mitral são as mais comumente afetadas, seguidas das válvulas tricúspide e pulmonar). A etiologia não é clara, sendo a hipótese de formação de microtrombos nas margens de coaptação das válvulas a mais aceite. Na ecocardiografia apresentam aspeto pediculado, móvel, com superfície filamentosa, tipicamente com uma aparência pontilhada nas margens e ecolucente. Do ponto de vista clínico, podem estar associados a fenómenos embólicos, no entanto, na maioria dos casos o diagnóstico é incidental. Apresentamos de seguida quatro casos de diagnóstico incidental de fibroelastomas nas quatro válvulas cardíacas, diagnosticados por ecocardiograma transtorácico (ETT) (Vídeo 1; Figura 1).

**Vídeo 1:**
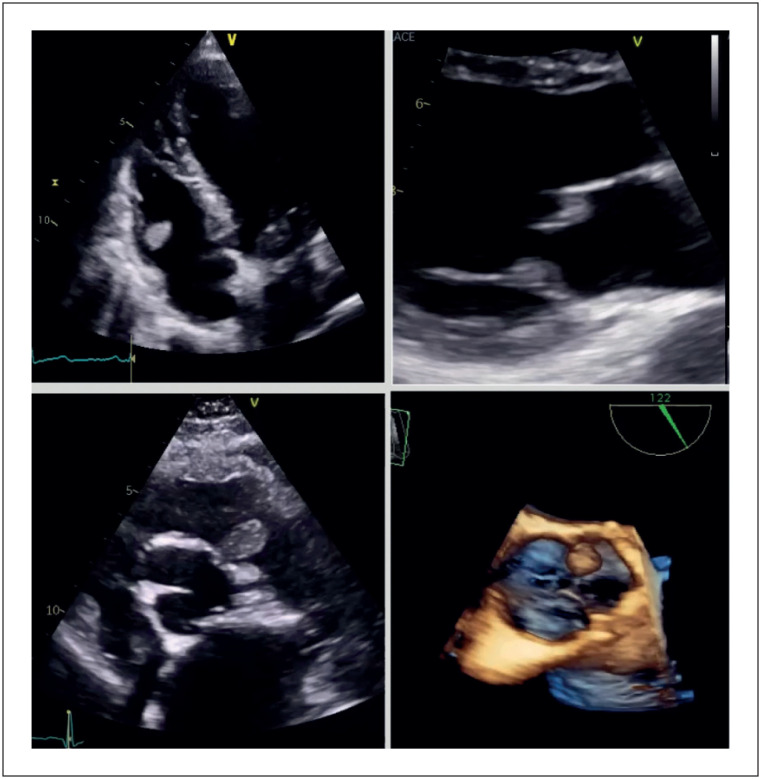
Da esquerda para a direita, de cima para baixo: fibroelastomas no folheto anterior da válvula tricúspide, folheto anterior da válvula mitral, cúspide esquerda da válvula pulmonar e cúspide esquerda da válvula aórtica, cada um correspondendo a um doente diferente. Em: http://abccardiol.org/supplementary-material/2024/12102/2023-0222_IM_video01.mp4

**Figura 1:**
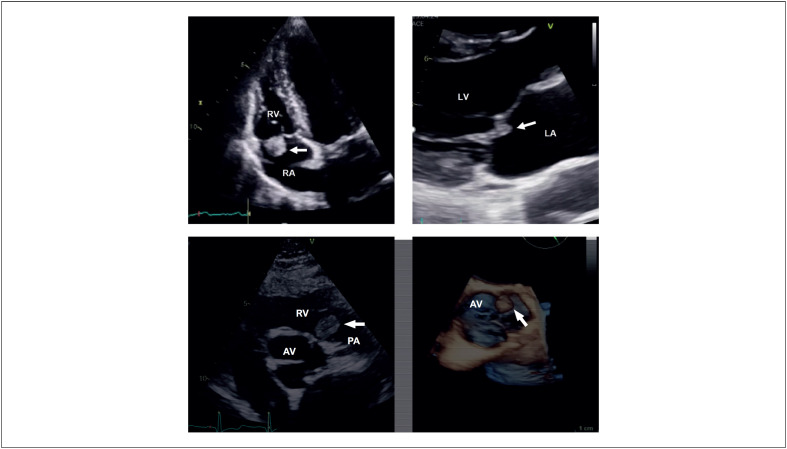
Da esquerda para a direita, de cima para baixo: fibroelastomas no folheto anterior da válvula tricúspide, folheto anterior da válvula mitral, cúspide esquerda da válvula pulmonar e cúspide esquerda da válvula aórtica, cada um correspondendo a um doente diferente.

## Descrição e Discussão

Apresentamos quatro casos de fibroelastomas diagnosticados por ecocardiografia transtorácica, cada um numa válvula cardíaca distinta.^1,2^ Em todos os casos, o achado ecocardiográfico foi incidental, em contraste com o típico diagnóstico que surge na sequência da investigação de um fenômeno embólico.^3,4^ Uma caracterização mais detalhada das estruturas foi obtida com o ecocardiograma transesofágico (ETE), confirmando a sua forma, bem como o pedículo que os unia à válvula cardíaca, a elevada mobilidade e aparência pontilhada, esta última correspondendo às projeções papilares encontradas na superfície. Nesta fase, o diagnóstico diferencial torna-se mandatório, nomeadamente com vegetação (recente ou antiga/calcificada) ou com trombo. Todavia, existiam pistas ecocardiográficas decisivas que apontavam para a hipótese de se tratar de fibroelastomas: (1) a forma oval ou arredondada dos fibroelastomas, (2) a sua aparência bem delimitada e homogênea (*vs* a aparência heterogénea das vegetações ou dos trombos), e (3) o aspeto pontilhado ao longo do perímetro (que não ocorre nas vegetações nem nos trombos). Adicionalmente, do ponto de vista clínico, a ausência de febre, parâmetros inflamatórios ou qualquer sinal ou sintoma sugestivo de quadro infecioso, tornam improvável a hipótese diagnóstica de endocardite infeciosa / vegetações no ecocardiograma.

Nos quatro casos apresentados, a localização, forma (em todos os casos redonda/oval, regular e com aspeto homogéneo), tamanho (3 a 8mm) e caracterização detalhada fornecida pelo ETE permitiram o diagnóstico presuntivo como fibroelastomas.^1^ Não se verificaram diferenças significativas entre fibroelastomas do coração direito *vs* coração esquerdo. Segundo a literatura, tais diferenças nunca foram documentadas. A tomografia computorizada cardíaca e a ressonância magnética cardíaca são modalidades de imagem igualmente úteis, particularmente para avaliação de válvulas do coração direito, onde a caracterização por ETT ou ETE pode ser difícil. Ainda assim, por limitações relacionadas com a resolução temporal, os fibroelastomas de menores dimensões podem permanecer indetectáveis.

Atualmente, não existem diretrizes dirigidas ao tratamento dos fibroelastomas. Segundo a literatura mais recente, é aceite que os fibroelastomas sintomáticos devem ser removidos cirurgicamente.^5^ Já em relação aos assintomáticos, as opiniões divergem e baseiam-se na mobilidade da massa e, consequentemente, no seu risco embólico, posição, necessidade de cirurgia cardíaca por outro motivo adicional e comorbilidades do doente.^6^ O tamanho e mobilidade da massa constituem os preditores independentes mais importantes de eventos embólicos, independentemente da localização no coração esquerdo ou direito. Os quatro casos foram discutidos em *Heart Team* e a remoção cirúrgica foi considerada o melhor curso de ação. A análise histológica que se seguiu confirmou o diagnóstico de fibroelastomas em todos os casos.

Nos quatro casos, a cirurgia cardíaca decorreu sem complicações. Não se registaram eventos embólicos nos pacientes após 2 anos de follow-up. Do ponto de vista ecocardiográfico, a morfologia das válvulas cardíacas manteve-se preservada, sem insuficiência ou estenose, e não houve recorrência da massa.

Os fibroelastomas são tumores benignos relativamente comuns, que podem afetar diferentes partes do coração, com especial ênfase nas quatro válvulas cardíacas. Afetam o funcionamento da válvula e são uma fonte de embolização. Não existem, atualmente, diretrizes para o tratamento dos fibroelastomas. Nos pacientes sintomáticos, a remoção cirúrgica é aconselhada. Nos assintomáticos, uma decisão partilhada deve tomar lugar, baseada nas características da massa, riso embólico e risco cirúrgico do doente.
