# Supplementary material for: Fibroelastoma, uma Doença Incidentaloma? – Imagem de Casos de Fibroelastomas como Achados Incidentais em Quatro Pacientes, Quatro Valvas Diferentes
Source: Arq Bras Cardiol. 2024 Feb 16;121(2):e20230222. [Article in Portuguese] doi: 10.36660/abc.20230222 (PMC11081129; doi:10.36660/abc.20230222)
Supplement: Translation

# Translation: Fibroelastoma, an Incidentaloma Disease? – Image Cases of Fibroelastomas as Incidental Findings in Four Patients, Four Different Valves

**Authors:** Joana Lima Lopes, Antonio Freitas, João Bicho Augusto

**Affiliations:** 1 Amadora Portugal Hospital Prof. Doutor Fernando Fonseca, Amadora – Portugal

**Keywords:** Fibroelastoma, Incidental Findings, Heart Neoplasias/surgery, Heart Valves, Echocardiography, Transesophageal/methods, Embolization

## Abstract

Fibroelastomas are the second most common benign cardiac tumor^1^. They are small avascular structures with a mean size of 9mm, ranging up to 70mm, usually attached to the heart valves’ surface (aortic and mitral are the most affected, followed by tricuspid and pulmonary valves). Their etiology is unclear, but the hypothesis of coalescence of microthrombus at the coaptation margins of valves is the most widely accepted theory. On echocardiography, they are pedicled, mobile, with a filamentous surface, and usually have a speckled appearance with echolucencies and a stippled pattern near the edges. Clinically, they may be associated with embolic phenomena; however, in most cases, the diagnosis is incidental. We present a series of four clinical cases with an incidental diagnosis of fibroelastomas across the four cardiac valves as assessed by transthoracic echocardiography (Video 1; Figure 1).

**Video 1:** From left to right and top to bottom: fibroelastomas of the anterior leaflet of the tricuspid valve, anterior leaflet of the mitral valve, left cusp of the pulmonary valve and left cuspid of the aortic valve, each corresponding to a different patient. Link: http://abccardiol.org/supplementary-material/2024/12102/2023-0222_IM_video01.mp4

**Figure 1:** From left to right and top to bottom: fibroelastomas of the anterior leaflet of the tricuspid valve, anterior leaflet of the mitral valve, left cusp of the pulmonary valve and left cuspid of the aortic valve, each corresponding to a different patient.

## Description and Discussion

We present four fibroelastomas that were first diagnosed by transthoracic echocardiography, each located in a different cardiac valve.^1,2^ In all cases, the echocardiographic finding was incidental as opposed to a diagnosis that is typically ascertained following an investigation of an embolic phenomenon.^3,4^ Further characterization of the masses with transesophageal echocardiography (TEE) was obtained, confirming their shape along with a pedicle attaching them to a heart valve, high mobility, and speckled appearance, the latter corresponding to the papillary projections found on the surface. At this stage, differential diagnosis is mandatory, namely with vegetation (new or calcified/ancient) or thrombus. However, crucial echocardiographic clues that pointed towards fibroelastoma were (1) the round or oval shape of fibroelastomas, (2) the well-demarcated and homogenous appearance (vs. heterogenous appearance of vegetation or thrombus), and (3) the speckled appearance with stippling around the perimeter (which does not exist in vegetations or thrombi). Additionally, from a clinical point of view, the absence of fever, inflammatory parameters, or any sign or symptom suggestive of an infectious condition makes the diagnostic hypothesis of infectious endocarditis/vegetation on the echocardiogram unlikely.

In the four cases presented, the location, shape (in all cases oval/round, relatively regular and homogenous appearance), size (3 to 8mm), and further detailed characterization by TEE allowed for a presumptive diagnosis of fibroelastoma.^1^ No relevant differences were found between right heart vs left heart fibroelastomas. According to recent literature, such differences were never documented. Cardiac computed tomography and cardiac magnetic resonance could also be used, particularly for right heart valves where TTE or TEE can be rather difficult; however, owing to temporal resolution limitations, small fibroelastomas can be missed.

There are no current guidelines for the treatment of fibroelastomas. According to current literature, symptomatic fibroelastomas should be surgically removed.^5^ As for asymptomatic fibroelastomas, opinions diverge and are based upon the mass mobility/embolic risk, position, concomitant cardiac surgery for other reasons, and comorbidities of the patient.^6^ The size and mobility of the tumors are the most important independent predictors of embolic events, regardless of left or right heart location. All four cases were discussed in the Heart Team, and surgical removal was considered the best course of action. Histological analysis confirmed the diagnosis of fibroelastoma in all cases.

In all cases, cardiac surgery was performed without complications. No embolic events were ever registered after a two-year follow-up. On echocardiographic follow-up, the valves morphology remains normal, no valve insufficiency or stenosis were noted, and there was no recurrence of the cardiac mass.

Fibroelastomas are (relatively) benign masses that can affect different parts of the heart and, crucially, all four cardiac valves. They can affect valvular function and serve as embolization material. There are no current guidelines for the treatment of fibroelastomas. In symptomatic patients, surgical removal is advised. In asymptomatic patients, a shared decision should take place based on the characteristics of the mass, embolic risk, and surgical risk of the patient.
